# Improved Bound Fit Algorithm for Fine Delay Scheduling in a Multi-Group Scan of Ultrasonic Phased Arrays

**DOI:** 10.3390/s19040906

**Published:** 2019-02-21

**Authors:** Yuzhong Li, Wenming Tang, Guixiong Liu

**Affiliations:** 1 School of Mechanical & Automotive Engineering, South China University of Technology, Guangzhou 510641, China; melyz@mail.scut.edu.cn (Y.L.); tang.wm@mail.scut.edu.cn (W.T.); 2 School of Information Engineering, Huizhou Economic and Polytechnic College, Huizhou 516057, China

**Keywords:** ultrasonic phased array, scheduling algorithm, multi-group sensors, FPGA

## Abstract

Multi-group scanning of ultrasonic phased arrays (UPAs) is a research field in distributed sensor technology. Interpolation filters intended for fine delay modules can provide high-accuracy time delays during the multi-group scanning of large-number-array elements in UPA instruments. However, increasing focus precision requires a large increase in the number of fine delay modules. In this paper, an architecture with fine delay modules for time division scheduling is explained in detail. An improved bound fit (IBF) algorithm is proposed, and an analysis of its mathematical model and time complexity is provided. The IBF algorithm was verified by experiment, wherein the performances of list, longest processing time, bound fit, and IBF algorithms were compared in terms of frame data scheduling in the multi-group scan. The experimental results prove that the scheduling algorithm decreased the makespan by 8.76–21.48%, and achieved the frame rate at 78 fps. The architecture reduced resource consumption by 30–40%. Therefore, the proposed architecture, model, and algorithm can reduce makespan, improve real-time performance, and decrease resource consumption.

## 1. Introduction

Ultrasonic phased array (UPA) technology is an important nondestructive testing method that is widely used in aerospace, shipbuilding, port machinery, and nuclear energy. With its multiple-group scanning functionality and a large number of other elements, the multi-group scan UPA system can provide extended scanning flexibility and image contrast, increased focal law diversification, and high signal-to-noise ratio (SNR). Within the system, a number of filters in a given module determine the precision of fine delay. The higher the precision, the better the image resolution. Classical all-parallel fine delay modules require a lot of hardware resources, i.e., a multiplier, look-up table (LUT), register (Reg), and an in field programmable gate array (FPGA). Synchronization and integration difficulty need to be considered in the use of multi-chip schemes, while hardware resources are limited in single chip schemes. Therefore, an architecture with time-division multiplexing is used to schedule frame tasks between fine delay modules in a single chip. This method can significantly improve resource utilization and reduce the number of resources used. However, when the sampling depth or the value of the focal law is large, the frame rate (frames per second, fps) decreases, leading to worse real-time performance of the distributed UPA instrument and a greatly reduced application scope. Therefore, it is necessary to coordinate fine modules and frame tasks for multi-group scanning through algorithm schedules, minimize idle time slots of resources in the fine delay modules, and reduce the makespan of all frame tasks to improve time performance.

In order to reduce the trade-off between the real-time processing of big data and system complexity, many studies have been conducted on high-performance hardware architecture and corresponding algorithms. Thus, various resource optimizations have been proposed. Holmes et al. [1] proposed a UPA system called the full matrix capture and total focus method (FMC-TFM), which requires the processing of large focus and delay data, and has been the mainstream architecture for research in recent years. Njiki el al. [2] proposed a hardware architecture for big data processing based on FMC and a large-scale phased array instrument, applied in the M2M NDT (nondestructive testing) (Eddyfi Technologies, Québec, QC, Canada) UPA system. The proposed FMC-TFM architecture can achieve a frame rate of 73.6 fps and 128 × 128 pixels in the region of interest. Shao and Yuan [3] proposed a method based on the compute unified device architecture (CUDA) interface, a parallel graphics processing unit (GPU), and whole parallel echo signal processing, wherein parallel GPUs accelerate the method 2–3-fold compared to MATLAB (Mathworks, Co., Ltd., Natick, MA, USA), while the multithreading CPU provides four times higher acceleration than a single thread. Guo et al. [4] improved a TFM imaging system and proposed an algorithm based on read-only memory (ROM). Zhang et al. [5] used a state machine, operation unit, and a large data storage unit to form the TFM algorithm imaging system, which achieved good performance. Tang et al. [6] proposed a data transmission algorithm for UPAs, but it does not work with delay and focus. Liu et al. [7] proposed an improved 8× interpolation cascaded integrator-comb (CIC) filter parallel algorithm, which reduced 12.5% of addition and 29.2% of multiplication and yielded a time delay accuracy of 1 ns at 125 MHz. Su et al. [8] proposed a parallel delay multiply and sum beamforming (PDMAS) algorithm, based on a graphics processing unit (GPU) that improved the parallelism and stability of the beamformer with a frame rate of 83 fps. However, these papers only focus on the performance of the delay and focus module, and not the multi-group scan and its frame task scheduling.

Although a multi-core CPU with single instruction multiple data (SIMD) and the GPU programmed by CUDA also realizes the beamform function (delay and focus), Asano et al. [9] found that a GPU was slower than a CPU for complex algorithms. Furthermore, they also found that a GPU only has potential for naïve computation methods, due to its small local memory and the memory access limitation in the architecture. The performance of a quad CPU is 1/12 to 1/7 that of a field programmable gate array (FPGA). The performance of an FPGA is only limited by its size and bandwidth. FPGA is the mainstream solution for portable UPA instruments, and is supported by manufacturers. Moreover, it is convenient for design and verification of the UPA system’s integrated circuits. Therefore, this paper uses FPGA to implement the algorithm and architecture of the multi-group scan UPA system.

The fine delay scheduling problem in the multi-group scanning of UPA systems, which we address here, can be considered as a parallel machine scheduling problem. The aim is to decrease the makespan, which can be represented as P_m_||C_max_. It is a non-deterministic polynomial-time hard (NP-hard) problem [10], which cannot be solved using polynomial algorithms. Heuristic algorithms are a simple and effective method used to address NP-hard problems at present.

The most commonly used heuristic algorithms are the longest processing time algorithm (LPT) [11] and the MULTIFIT algorithm. The MULTIFIT algorithm proposed by Coffman et al. [12] is based on the first fit decreasing (FFD) iteration algorithm, which is used in bin-packing problems. However, the MULTIFIT algorithm has much better performance than the LPT algorithm. Freisen et al. [13] studied the absolute performance and time complexity of the MULTIFIT algorithm. Lee et al. [14] used a combination of the LPT and MULTIFIT algorithms. Kang et al. [15] simplified the MULTIFIT algorithm and combined it with the prepare algorithm (PA), in order to form the bound fit (BF) algorithm. Li et al. [16] proposed the QUICKFIT algorithm, which is an improved BF algorithm in the iteration stage. Based on the advantages of the LPT and BF algorithms, the improved bound fit (IBF) algorithm is proposed here.

In this paper, a fine delay scheduling architecture was also analyzed considering multi-group-scan echo data diversity, using a non-preempt model for the scheduling problem and proposing the IBF algorithm for optimization. 

The paper is organized as follows. In Section 2, the architecture of the fine delay module scheduling for the multi-group scanning of UPA systems is presented, and the multi-group scan problem is explained. In Section 3, the IBF algorithm is proposed and an analysis of its performance and time complexity is provided. LIST, LPT, BF, and IBF algorithms are compared in Section 4. Finally, a conclusion is provided in Section 5.

## 2. Fine Delay Module for Multi-Group Scanning of UPAs

### 2.1. Fine Delay Scheduling Principle

The delay method and focus scheduling based on different UPA instrument focal parameters (e.g., number of apertures, sending and receiving time, and data amount), which control the pulse repetition frequency (PRF) and frame formation, are used for scheduling in multi-group scans. The delay precision is 1.25 ns. Due to the limitation of the resources of the FPGA in our experiments, the system architecture is designed as four groups and two fine delay modules. Each group has eight channels, and each channel has 10-bit analog-digital converter (ADC). Sampling depth is 2–8 K, the number of focal law ≤128, and read parameter length is 1024 in each group. The design frame rate is not less than 24 fps, which meets the requirements of real-time display.

**Figure 1:**
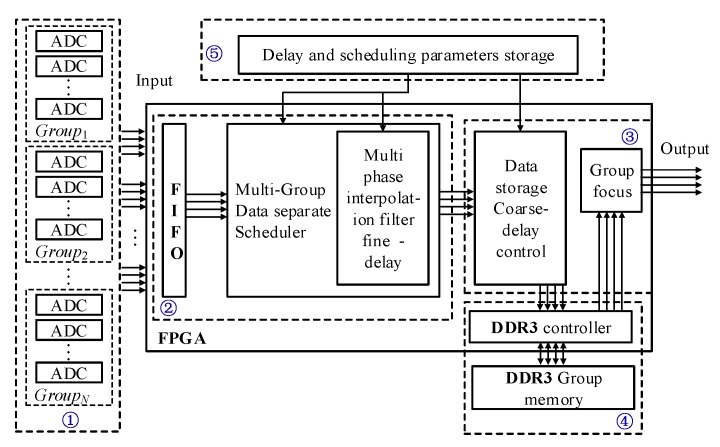
Diagram of the fine delay module for multi-group scanning.

A diagram of the for mutli-group scanning is shown in Figure 1, labels ①–⑤ in Figure 1 are described below.

The presented block diagram includes the following parts:

(1) High speed multi-channel ADC module (HADC): Ultrasonic echo signals are subjected to high-speed multi-channel ADC acquisition, conditioning conversion, and transformation into low-voltage differential signaling (LVDS) serial signals. They are then fed to the FPGA for further processing. ADCs are divided into groups according to the probe socket and multi-group scan.
(2) Fine delay scheduling module (FDS): The LVDS serial signal is first converted into a parallel signal, then the parallel signal generated by the IP core is sent to the multi-channel first-in first-out memory (FIFO), which is used for buffering and scheduling. The scheduling module consists of several fine delay modules. The signal buffered in the FIFO is then fed to the scheduling module, where it is forwarded to different fine delay modules. Thus, time division multiplexing is achieved.

**Figure 2:**
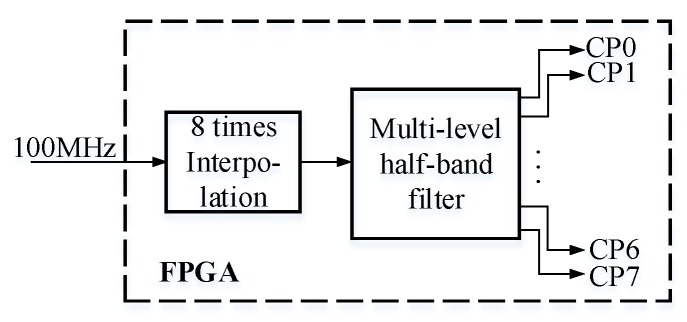
Diagram of fine delay module.

**Figure 3:**
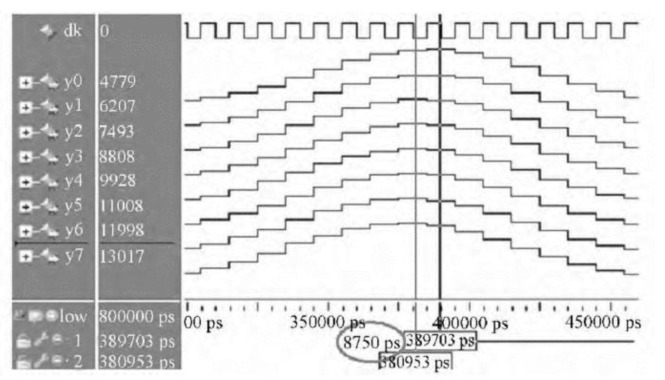
ModelSim simulation diagram of the multi-level half-band fine delay filter.

The fine-delay module used in this study contains the multi-level half-band filter that was proposed by Liu and Tang [17]. A diagram of the multi-level half-band fine delay filter is presented in Figure 2, whereas its simulation diagram created in ModelSim (Mentor Co., Ltd., Wilsonville, OR, USA) is shown in Figure 3.

The multi-level half-band fine delay filter uses the interpolation method with eight time intervals to design a half-band filter. The implementation of synthetic technology in the multi-level half-band interpolation filter results in filter decomposition into eight sub-filters. Simultaneously, interpolation with poly-phase decomposition is achieved. The eight filters delay the original signal for 0, 1.25, 2.5, 3.75, 5, 6.25, 7.5, and 8.75 ns. The data samples have a 10-bit length, and thus two 9-bit multipliers are needed for multiplications. However, the multi-level half-band filter uses six 9-bit multipliers. In addition, each channel has eight fine delay channels, so there are 96 (i.e., 6 × 2 × 8 = 96) 9-bit multipliers. If all parallel delay is used in a 256-element UPA system, then 24,576 multipliers would be needed. Given such large resource consumption, the integration of a single FPGA in the multi-group scan module of a UPA system would be difficult.

**Figure 4:**
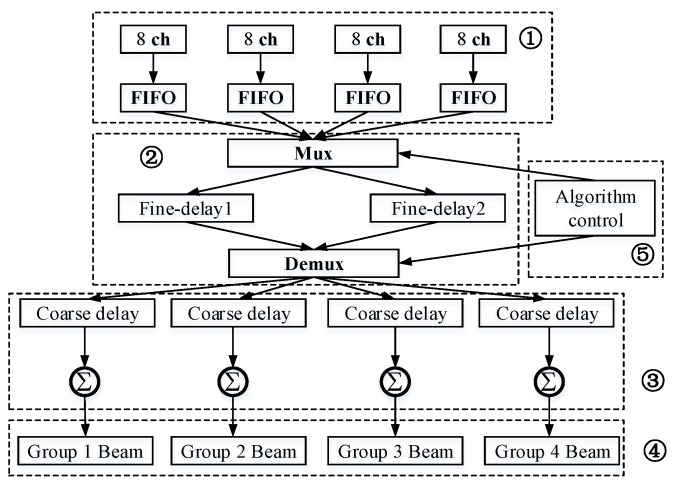
Fine delay scheduling model diagram in the multi-scan group.

(3) Coarse delay and sum module (CDS): Coarse delay is based on counter clock delay technology. All the relative delay parameters of focal laws, calculated by a PC, can be loaded from the “delay and scheduling parameters storage” block in Figure 1. The double data rate 3 (DDR3) synchronous dynamic random access memory input signal addresses the corresponding coarse delay parameter counted by the clock, and thus fixed integer coarse delay is achieved. The sum module merges signals processed by fine delay and coarse delay blocks in an ultrasonic digital beam, which represents the complete beamform of the focal laws. All signals of the ultrasonic digital beam are stored in memory, and all signal groups form a corresponding beamform. In other words, each focal law forms a digital beamform, and all the beamforms of the same group generate the initial image information of that group.
(4) External DDR3: Since the internal RAM capacity of the FPGA is insufficient, a DDR3 controller with two DDR3 memories is used for coarse delay data storage. DDR3 memory has a coarse delay and reads the group focus module according to the group.
(5) Delay and scheduling parameters storage (DSPS): Delay and scheduling parameters storage is a large-scale storage block in the FPGA. The delay and scheduling parameters are calculated using a focal law calculator in the PC, corresponding to the input data entered by the user. DSPS contains a scheduling table, the pulse repetition frequency of each group, and the time delay parameter for both fine and coarse delays according to focal laws. It also includes algorithmic control for scheduling Mux and Demux based on the above parameters. A fine delay scheduling model diagram in the multi-scan group is presented in Figure 4.

### 2.2. Fine Delay Scheduling Problem in Multi-Group Scanning

The parameters of the fine delay module for multi-group scanning of UPAs are presented in Table 1. Here, we represent the symbols used in the scheduling problems with brackets.

Fine-delay scheduling for multi-group scanning of UPAs must satisfy four conditions:

(1) Each focal law must be separately processed in fine delay modules. In other words, one fine delay module must process only one focal law datum.
(2) The process cannot be interrupted or preemptive, i.e., a no-interrupt non-preemptive (NINP) model is adopted.
(3) There is no time gap between the start time of focal law and the start time of the pulse repetition period.
(4) The sample depth is less than the pulse repetition period.

Condition (1) avoids timing confusion, condition (2) avoids interruption of the fine delay signal processing, and condition (3) compacts the frame task for scheduling and decreases the time slot waste. Condition (4) ensures that the fine delay processing will not exceed its abilities, leading to echo data overlap. 

Before a description of the fine delay scheduling problem is presented, some parameters must be defined:

**Definition** **1.** *Frame task.*

If it is assumed that the *i*th scan has focal law frame $N_{\mathrm{FocalLaw}}^{i}$ and sample depth $D_{\mathrm{Sample}}^{i}$, then the frame task is the time needed to complete all beamforms (or focal laws) of the image.

**Definition** **2.** *Frame task deadline.*

The frame task deadline represents the time the system needs to generate a complete image for all groups, and it must be less than 1/24 s for real-time applications. 

**Figure 5:**
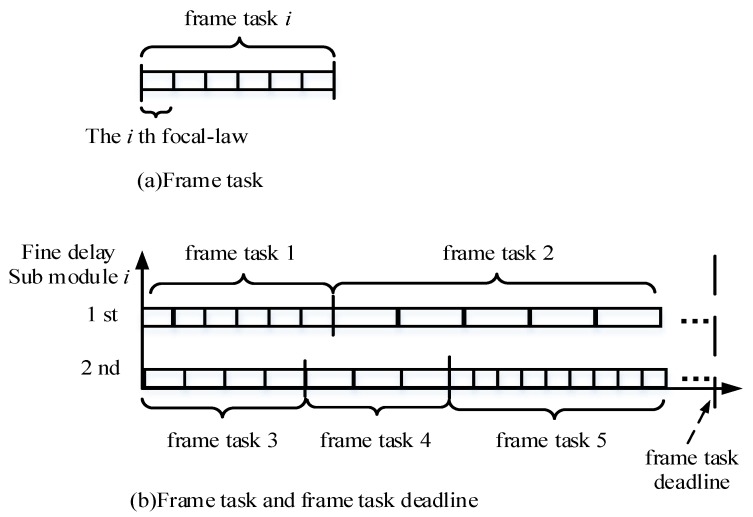
Schematic diagram of: (**a**) Frame task and (**b**) Frame task and frame task deadline.

Schematic diagrams of the frame task and frame task deadline are presented in Figure 5a,b, respectively.

The time parameters used in the proposed algorithm are defined as follows.

Start time, $t_{s}^{i}$, is defined by:

(1) $$t_{s}^{i}=0\;i=1,2,\ldots ,N_{\mathrm{Group}}$$

Processing time, $t_{p}^{i}$, is defined by:

(2) $$t_{p}^{i}=(D_{\mathrm{sample}}^{i}\times T_{\mathrm{clock}-\mathrm{cycle}}+T_{\mathrm{RP}}^{i})\times N_{\mathrm{FocalLaw}}^{i}\;i=1,2,\ldots ,N_{\mathrm{Group}}$$

End time, $t_{d}^{i}$, is defined by:

(3) $$t_{d}^{i}=1/24\;s\;i=1,2,\ldots ,N_{\mathrm{Group}}$$

Therefore, the question can be set as $P_{m}||C_{\max}$, and the scheduling model is defined by:

(4) $$Min\;z\;=\;Max(\sum_{j=1}^{n}t_{p}^{j}x_{ij})\;i=1,2,\ldots ,m$$

subject to:

(5) $$\sum_{j=1}^{n}t_{p}^{j}x_{ij}\leq t_{d}y_{i}\;i=1,2,\ldots ,m\;j=1,2,\ldots ,n$$

(6) $$\sum_{i=1}^{m}x_{ij}=1\;i=1,2,\ldots ,m\;j=1,2,\ldots ,n$$

(7) $$x_{ij}\in \{0,1\}$$

(8) $$t_{d}\leq 1/24$$

Equation (4) refers to the scheduling goal of minimizing the project’s maximum completion time, which represents the time needed for the completion of all project tasks. In this paper, we consider the frame task as the job or task of the scheduling problem. According to Equation (5), the time allocation of each fine delay module cannot be greater than *t_d_*. Equations (6) and (7) show that any task can be assigned only to one processor, and *x_ij_* is an assigned variable that is equal to zero or one. Equation (8) represents all fame tasks that must be finished before the frame task deadline. 

## 3. IBF Algorithm

Since there is no dependency between tasks, the fine delay scheduling problem in multi-group scanning can be considered as an independent, parallel processor scheduling task. 

The IBF algorithm parameters are defined as follows. Input is the set of tasks *T* = {*t_i_*, *i* = 1,2,…,*n*}, the number of fine-delay modules is *m*, and the number of tasks is *n*. Output is the maximal processing time, $C_{\max}^{\mathrm{IBF}}$.

The IBF algorithm steps are as follows:

Step 1. Sort tasks *T* in descending order according to the task processing time: *p_i_*, *i* = 1,2,…,*n*;

Step 2. Assume that $A=\frac{1}{m}\sum_{i=1}^{n}p_{i}$ and $L_{j},\;j=1,2,\ldots ,m$ are the focus and delay module pointers, respectively;

Step 3. Use the LPT algorithm to obtain the maximal processing time $C_{\max}^{\mathrm{LPT}}$. Let *l* = 1 and $B(1)=C_{\max}^{\mathrm{LPT}}$;

Step 4. If *A* < max(*L_j_*) < *B*(*l*), go to step 5; otherwise, go to step 8;

Step 5. Let *l* = *l* + 1, *i* = 1, *B*(*l*) = min(max(*L_j_*), *B*(*l* − 1) − 1);

Step 6. If there is at least one *j* that satisfies the condition *L_j_* + *p_i_* ≤ *B*(*l*), then allocate task *t_i_* to the focus and delay module, which satisfies condition *L_j_* + *p_i_* ≤ *B*(*l*). Otherwise, allocate the task to the focus and delay module, which provides the minimal value of *L_j_* + *p_i_*;

Step 7. Set *i* = *i* + 1, and if *i* ≤ *n*, go back to step 6; otherwise, go back to step 4;

Step 8. $C_{\max}^{\mathrm{IBF}}=\min(B\left(1\right),B\left(2\right),B(l-1))$.

**Figure 6:**
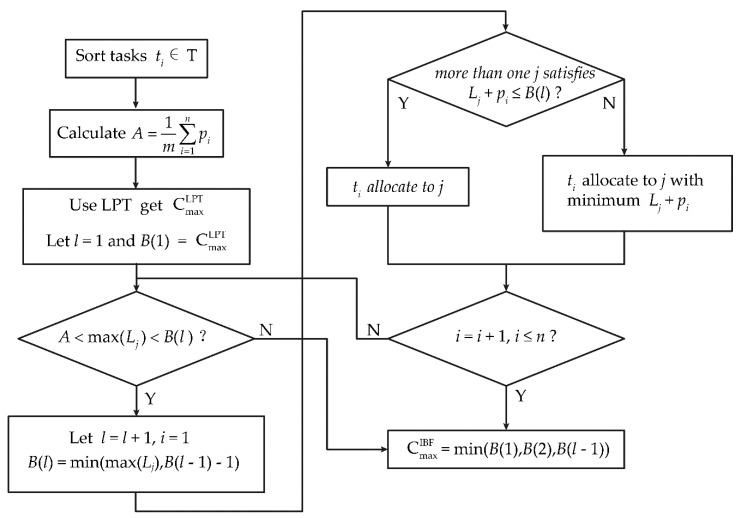
Improved bound fit algorithm (IBF) flowchart. LPT: longest processing time algorithm.

In step 3, the LPT algorithm is used to calculate the initial processing time in order to better approximate the initial conditions. Steps 4–8 represent the prepare algorithm (PA). Thus, the IBF algorithm is a combination of LPT and PA that improves the boundary and convergence of iteration, and achieves better performance in terms of local search and iterative progression. The IBF flowchart is shown in Figure 6.

The IBF algorithm analysis is obtained for $B(1)=C_{\max}^{\mathrm{LPT}}$. In the case the iteration stops at *l* = 2, then the output algorithm result will be $C_{\max}^{\mathrm{IBF}}=C_{\max}^{\mathrm{LPT}}$. If the iteration stops at *l* = 3, then the output result will be $C_{\max}^{\mathrm{IBF}}=C_{\max}^{\mathrm{PA}(B(0))}$, and that wil be the makespan. If the iteration stops at *l* > 3, then $C_{\max}^{\mathrm{IBF}}=C_{\max}^{\mathrm{PA}(B(l-1))}$. 

From *B*(*l*) = min(max(*L_j_*), *B*(*l* − 1) − 1), we obtain *B*(*l*) ≤ *B*(*l* − 1) − 1. Thus, *B*(2) ≤ *B*(1) − 1, *B*(3) ≤ *B*(2) − 1 ≤ (*B*(1) − 1) − 1 = *B*(1) − 2.

After induction *B*(*l*) ≤ *B*(1) − (*l* − 1). Therefore, the absolute performance of the IBF algorithm is defined by:

(9) $$C_{\max}^{\mathrm{IBF}}\leq \left(\frac{4}{3}-\frac{1}{3m}\right)C_{\max}^{\mathrm{OPT}}-(l-1)$$

If the iteration number is equal to one, the IBF time complexity is defined by:

(10) O(nlogn+nllogm)

If the number of iterations is greater than one, IBF employs the PA, which represents the FFD algorithm used in the bin-packing problem. 

## 4. Experimental Results

### 4.1. Time Performance

In order to determine the real-time performance of the IBF algorithm, a randomly generated set of tasks was used. The set and real-time deadline were used to simulate a UPA multi-group fine delay scheduling problem. The specific task generation process was as follows. First, *m* time blocks were generated. The length of each time block was as long as the deadline *t_d_*. Then, each task block was divided into h=⌈n/m⌉+1 parts, and thus *h × m* tasks were obtained in *m* time blocks. Afterward, *n* tasks from *h × m* tasks that were generated from the previous step were chosen to create a set of tasks, and all task lengths were multiplied by 0.99. Thus, a random generation of a set of tasks was produced. The whole experiment ran in I7-4850HQ (Intel Corporation, Santa Clara, CA, USA) 8 GB RAM with MATLAB 2016a.

This process was conducted to ensure that the processing time of each generated task was not greater than the real-time deadline. All generated tasks did not exceed the calculating ability of the fine-delay module. In other words, a feasible solution always existed for a given scheduling in terms of the number of modules that satisfied the required conditions. The generated set was subjected to a random uniform distribution, and a variety of large scopes were covered. 

Five tests were conducted with the following parameters: the number of fine-delay modules *m*, the ratio of number of tasks and fine delay modules *k* = *n*/*m*, the real-time deadline *d*, the number of iterations *K*, and makespan *C_max_*. Each test was generated 100 times, and the average result was calculated. The LIST, LPT, BF, and IBF algorithms were compared.

**Figure 7:**
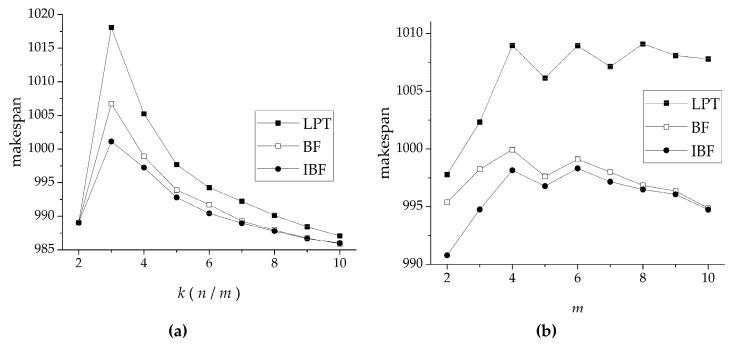
Comparison of LPT, bound fit (BF), and IBF in terms of makespan with (**a**) variable *k* (ratio of the number of tasks *n* and the number of fine delay modules *m*) and (**b**) variable number of fine delay modules *m*.

Test 1 compared LPT, BF, and IBF algorithms in terms of makespan. In Figure 7a, the parameter settings were: *m* = 4, *k* = 2–10, and *d* = 1000. Note that each curve had a peak value at *k* = 3, because when *k* = 3, the method generating the problem reduced the number of tasks and increased the length. Under this condition, the problem was difficult to schedule. With gradually increasing *k*, all curves gradually declined. IBF had the smallest makespan at *k* < 8, and when *k* ≥ 8, IBF and BF almost had the same makespan performance. This is because with the increase in *k*, the problem produced more tasks and the length decreased. That is, the smaller the granularity of the tasks, the greater the role of the scheduling algorithm. In Figure 7b, the parameter settings were: *m* = 2–10, *k* = 4, and *d* = 1000. We can see that the IBF algorithm still had the smallest makespan, but with the increase in *m*, the gap between BF and IBF continued to narrow. Although *k* was unchanged, the larger the value of *m*, the greater the permutations and combinations of the scheduling algorithm were. In makespan comparisons, IBF always had the best performance, but, as parameters *k* and *m* increased, the performance of BF and IBF gradually approached each other.

**Figure 8:**
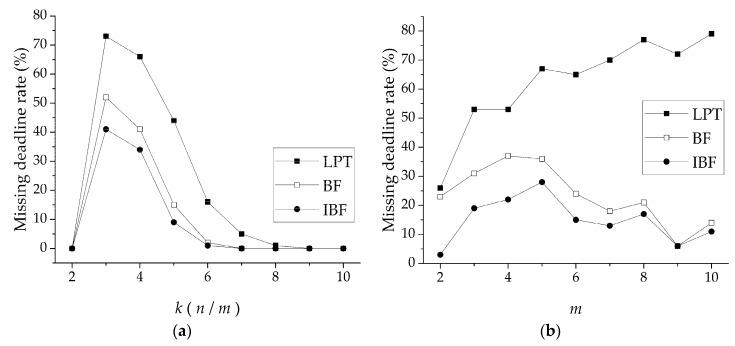
Comparison of LPT, BF, and IBF in terms of missed deadline rate (MDR) with (**a**) *k* (the ratio of the number of tasks *n* and the number of fine-delay modules *m*) and (**b**) variable number of fine delay modules *m*.

Test 2 compared LPT, BF, and IBF in terms of the missed deadline rate (MDR) with variables *k* and *m*. The parameter settings in Figure 8a were the same as in Figure 7a, and those in Figure 7b were applied to Figure 8b. The MDR is defined as the number of times a deadline was missed when a scheduling problem was generated randomly 100 times. Figure 8a shows that all curves had a peak value at *k* = 3, and then gradually decreased with increasing *k*. The reason is similar to test 1. Note that in Figure 8b, IBF had the smallest makespan, but when *m* > 9, the values of BF and IBF were basically the same. IBF was still the best in MDR performance, and with the increase in *k*, the scheduling performance improved as well. When *k* > 8, IBF was not significantly superior to BF.

**Figure 9:**
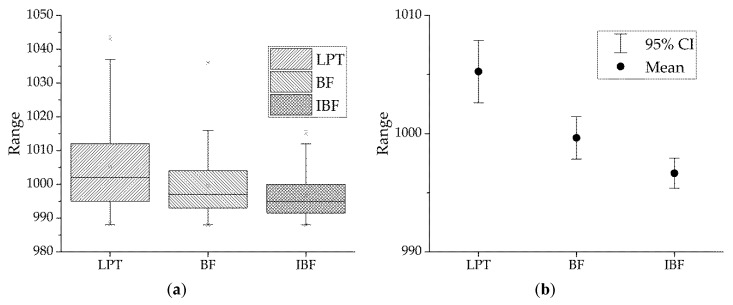
Comparison of LIST, LPT, and IBF algorithms in (**a**) boxplot and (**b**) 95% confidence interval (CI) plot.

Test 3 compared LPT, BF, and IBF using statistical plots. Parameter settings were *m* = 4, *k* = 4, and calculation was run 100 times to obtain the makespan. Figure 9a shows the box plot. Note that the IBF algorithm had the lowest median and upper limits and the narrowest interquartile range (IQR). This shows that IBF scheduling had the best overall performance and the most centralized data. In the 95% confidence interval (CI) plot in Figure 9b, IBF had the lowest mean and the narrowest 95% CI. The IBF algorithm outperformed the BF and LPT algorithms in terms of statistical performance.

Test 4 compared the performance of LIST, LPT, BF, and IBF algorithms (Table 2). The test parameter settings were *m* = 4, *k* = 4, *d* = 1000, and the average of 100 runs was taken. The LIST algorithm had the worst performance, which affected the display of the figures. In order to clearly compare BF and IBF, which was not mentioned in the previous experiments, $R_{\mathrm{IBF}/\mathrm{LIST}}$ was defined as follows:

(11) $$R_{\mathrm{IBF}/\mathrm{LIST}}=\frac{\bar{C_{\max}^{\mathrm{LIST}}}-\bar{C_{\max}^{\mathrm{IBF}}}}{\bar{C_{\max}^{\mathrm{LIST}}}}\times 100\%$$

where $\bar{C_{\max}^{\mathrm{LIST}}}$, $\bar{C_{\max}^{\mathrm{LPT}}}$, $\bar{C_{\max}^{\mathrm{BF}}}$, and $\bar{C_{\max}^{\mathrm{IBF}}}$ represent the average makespans of LIST, LPT, BF, and IBF obtained from 100 runs, respectively. In addition, $\bar{K^{\mathrm{BF}}}$ and $\bar{K^{\mathrm{IBF}}}$ represent the average number of iterations for BF and IBF. As shown in Table 2, IBF had the lowest average makespan, but its average number of iterations was slightly greater than that of the BF algorithm. This was also reflected in the elapsed time. In the worst case of our experiment, the average elapsed times at *m* = 10, *k* = 4 for LIST, LPT, BF, and IBF algorithms were 2.70, 2.63, 40.61, and 55.21 ms, respectively. The elapsed time of IBF was greater than BF by about 35.95%. However, as shown in the last column of Table 2, IBF improved performance by 8.76–21.48% compared to the LIST algorithm.

**Figure 10:**
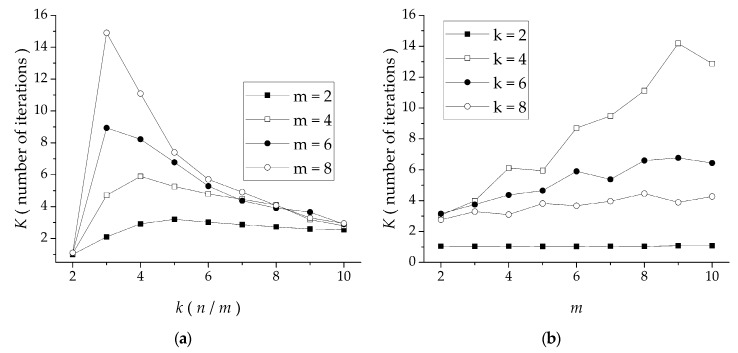
IBF number of iterations for: (**a**) *k* = 2–10 and (**b**) *m* = 2–10.

Test 5 was used to examine the relationship of IBF with the number of iterations. In Figure 10a, all curves had a peak value at *k* = 3–5, and then slowly declined. This occurred because when *k* = 35, the generated tasks had large granularity, which facilitated iteration without satisfying the conditions, so the number of iterations was greater. The number of iterations with larger *m* was greater than that with smaller *m*, because a large *m* leads to more permutations and combinations. When *k* > 8, the number of iterations decreased gradually and tended to be the same. Due to the small size of the task, the initial LPT algorithm was more effective, so the number of iterations decreased. In Figure 10b, except for the case of *k* = 2, the other curves increased gradually, and the larger the value of *k*, the smaller the number of iterations. Therefore, the greater the task granularity, the greater the value of *m* and the greater the number of iterations.

### 4.2. Resource Consumption

In the experiment, an Altera Cyclone VI EP4CE115F29C8 and Quartus II 13.0 (Intel Corporation, Santa Clara, CA, USA) were used to compare all parallel and 1/2 scheduling for 32-channel and 64-channel architectures. Then, the TimeQuest Timing Analyzer in Quartus II was used to determine the maximal clock frequency for the listed architectures. The clock frequency was set to 100 MHz. The obtained resource consumption and maximal frequencies of all architectures are presented in Table 3, wherein “number of groups” represents the number of scan groups in the multi-group UPA system; “number of modules” represents the number of fine delay modules in the system; “Total LUT” (LUT: look up table), “Total Reg.”, and “Total 9-bit Mult.” refer to the consumption of total logic unit, total register, and total 9-bit multiplier, respectively; and Fmax represents the maximum clock frequency. Percentages with brackets in the Total LUT and Total 9-bit Mult. columns represent their share of all the same resources in the entire FPGA.

Table 3 shows that all parallel architectures demand more resources and have lower maximal frequencies than 1/2 scheduling architectures. The 1/2 scheduling architecture could save about 57.06–58.84% in LUT and 30–40% in 9-bit multipliers. Table 3 also demonstrates that maximum frequency decreased as the number of channels increased. The bold text in column Fmax are the best Fmax in same number of channels, respectively. Therefore, based on the premise of guaranteeing real-time performance, the proposed architecture and IBF algorithm can reduce resource consumption, shorten timing, and increase the maximum clock frequency. 

### 4.3. Real-Time Verification

**Figure 11:**
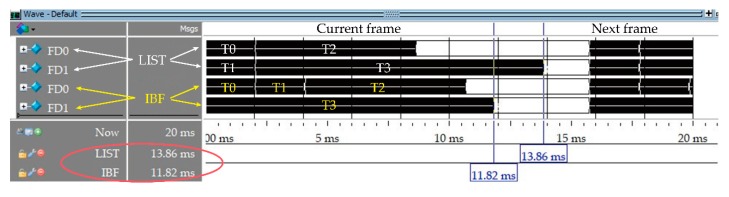
Four groups scheduled in two fine delay modules’ simulation by ModelSim.

Figure 11 displays the results of the pre-synthesis simulation in four groups of two fine delay modules, using ModelSim 10.2 SE electronics design automation tools (Mentor Co., Ltd., Wilsonville, OR, USA). The other experimental conditions are described in the previous section, and the experimental parameters are shown in Table 4. The delay caused by fine-delay filters with eight clock-cycles has been taken into account and combined into time of read parameter. Units are clock cycles of the FPGA in Table 4 columns 2–4.

In Figure 11, the tasks were T0–T3, corresponding to frame tasks of Group 0–3, and FD0 and FD1 are fine delay modules. The upper FD0 and FD1 were scheduled by LIST, and the lower FD0 and FD1 were scheduled by IBF. In the case of maximum 8 K sampling depth, 128 focal laws (Group 3), the makespan of LIST was 13.86 ms, whereas the makespan of IBF was 11.82 ms, so IBF is superior to LIST. At a waiting time of more than 1 ms between frames, the frame periods of LIST and IBF were 14.86 and 12.82 ms, respectively, which correspond to frame rates of 67 and 78 fps, respectively. Therefore, the IBF algorithm generally reduced the makespan of the frame tasks, increased the frame rate, and improved real-time performance of the multi-group scan UPA instrument.

## 5. Conclusions

In this paper, a fine delay scheduling architecture in the multi-group scanning of a UPA system was presented. The diversity of echo data in multi-group scanning and the number of focal laws were considered, and the multi-group scan problem was modelled by a linear equation. The IBF algorithm was proposed, and its time complexity and absolute performance were analyzed. The experimental results showed that compared to LIST, LPT, and BF algorithms, the IBF algorithm decreased the makespan by 8.76–21.48%, while the frame rate reached 78 fps, and the architecture reduced FPGA resources by 30–40%. The IBF algorithm was superior to BF in terms of its small task-to-module ratio. The proposed algorithm and mathematical model was applied to a UPA. uUsing the proposed architectures effectively improved integration, increased maximum frequency, improved real-time performance, and finally, decreased resource consumption. Therefore, the instrument’s flexibility and performance was improved. The next step is to study another processing module scheduling and multi-FPGA situation, integrated in a distributed environment.

## Figures and Tables

**Table 1:** Parameters of the fine delay module for multi-group scanning of a ultrasonic phased array (UPA) system.

Symbol	Parameter
$N_{\mathrm{Group}}$	Number of groups (*n*) ^1^
$N_{\mathrm{FocalLaw}}^{i}$	Number of focal laws in the *i*th group
$D_{\mathrm{Sample}}^{i}$	Sample depth of the *i*th group
$N_{\mathrm{FDModule}}$	Number of fine delay modules (*m*) ^1^
$T_{\mathrm{RP}}^{i}$	Read parameter time of focal law
*T*_clock-cycle_	Clock period in FPGA
$t_{p}^{i}$	Processing time in the *i*th group (*p_i_*) ^1^

^1^ Symbols in brackets are those used in the scheduling problem.

**Table 2:** Comparison of LIST, LPT, BF, and IBF performance.

*m*	LIST	LPT	BF		IBF		$R_{\mathrm{IBF}/\mathrm{LIST}}$
	$\bar{C_{\max}^{\mathrm{LIST}}}$	$\bar{C_{\max}^{\mathrm{LPT}}}$	$\bar{C_{\max}^{\mathrm{BF}}}$	$\bar{K^{\mathrm{BF}}}$	$\bar{C_{\max}^{\mathrm{IBF}}}$	$\bar{K^{\mathrm{IBF}}}$	
2	1092.39	996.67	995.96	3.02	991.17	2.64	8.76%
4	1174.41	1005.26	999.65	4.01	996.65	5.49	14.40%
6	1264.74	1007.05	997.98	5.28	996.91	8.11	20.37%
8	1258.18	1008.45	997.63	6.86	997.16	10.1	19.85%
10	1282.96	1007.41	996.22	8.01	996.06	11.61	21.48%

**Table 3:** Resource consumption and max frequency of all parallel and 1/2 scheduling for 32-channel and 64-channel architectures.

	Number of Groups	Number of Modules	Total LUT	Total Reg.	Total 9-bit Mult.	Fmax (MHz)
All-par. 32 ch.	4	4	5086 (4.44%)	3977	320 (60%)	137.74
1/2 Sch. 32 ch.	4	2	2902 (2.53%)	2445	160 (30%)	146.99
All-par. 64 ch.	8	8	14,340 (12.53%)	8569	532 (100%) ^1^	113.77
1/2 Sch. 64 ch.	8	4	5902 (5.16%)	4857	320 (60%)	126.53

^1^ Due to resource limitations, the total 9-bit multiplier in the FPGA was 532.

**Table 4:** Four groups of two fine delay modules simulation parameters.

Group	Number of Focal Laws $\left(N_{\mathrm{FocalLaw}}^{i}\right)$	Sample Depth $\left(D_{\mathrm{Sample}}^{i}\right)$	Processing Time ^1^ (*p_i_*)
0	64	2048	196,608
1	64	2048	196,608
2	128	4096	655,360
3	128	8192	1,179,648

^1^ Time of read parameters $T_{\mathrm{RP}}^{i}$ = 1024.
